# The Prognostic Value of Excision Repair Cross-Complementation Group 1 (ERCC1) in Patients with Small Cell Lung Cancer (SCLC) Receiving Platinum-Based Chemotherapy: Evidence from Meta-Analysis

**DOI:** 10.1371/journal.pone.0111651

**Published:** 2014-11-06

**Authors:** Yanlong Yang, Xiuping Luo, Nuo Yang, Ronghao Feng, Lei Xian

**Affiliations:** 1 Department of Cardiothoracic Surgery, The First Affiliated Hospital of Guangxi Medical University, Nanning, Guangxi Zhuang Autonomous Region, China; 2 Department of Chemotherapy, The Affiliated Tumor Hospital of Guangxi Medical University, Nanning, Guangxi Zhuang Autonomous Region, China; Duke Cancer Institute, United States of America

## Abstract

Recently, the correlation between the efficacy of platinum-based chemotherapy and ERCC1 expression in patients with SCLC has attracted wide-spread attention, and a lot of investigations have been conducted, whereas conflicting results were presented. Therefore, we performed the present meta-analysis of eligible studies to derive a more precise evaluation of the association between ERCC1 expression and the clinical outcome in SCLC patients receiving platinum-based chemotherapy. A literature search for relevant studies was conducted in the electronic databases of PubMed, EMBASE and Web of Science. The inclusive criteria were SCLC patients treated by platinum-based chemotherapy, and evaluated the relationship between ERCC1 expression and the clinical outcomes [including overall response rate (ORR), overall survival (OS) or progression-free survival (PFS)]. Odds ratio (OR) or hazard ratio (HR) with 95% confidence interval (CI) was calculated to assess the risk. A total of nine studies including 1129 patients were included in final analysis. Our analysis indicated that positive/high ERCC1 expression was associated with unfavorable OS (HR = 1.18, 95%CI = 1.02–1.37) and PFS (HR = 1.46, 95%CI = 1.14–1.88). Subgroup analysis according to disease stage suggested the significant relationship was found in limited stage (LS-SCLC), but not in extensive stage (ES-SCLC). However, no significant association was found between ERCC1 expression and ORR. Our analysis suggested ERCC1 expression may be a prognostic factor in SCLC patients receiving platinum-based chemotherapy, especially for LS-SCLC.

## Introduction

Lung cancer is a common malignancy and has been a leading cause of mortality and a burden of health finance cost worldwide [1]. It has been reported by the International Agency for Research on Cancer for 2008 that a total of 1.6 million individuals were diagnosed as lung cancer and approximately 1.4 million died from the disease, making it the first leading cause of cancer mortality in men and second in women throughout the world [2]. Accounting for around 14% of lung cancer new cases in the Europe, USA, and Korea, small cell lung cancer (SCLC) is the most aggressive subtype of lung cancer and is characterized by rapid growth and early dissemination [3]–[6]. Most of the SCLC cases (70%–80%) are diagnosed at an extensive stage (ES-SCLC) and only a small number of SCLC patients are found to be limited to the thorax (LS-SCLC) [7]. In fact, the median survival time is 14 to 20 months for LS-SCLC and 7 to 10 months for ES-SCLC [8]. The standard treatment for patients with limited stage is a combination of chest radiation and chemotherapy on the basis of etoposide and cisplatin. For patients with extensive stage, platinum-based combination chemotherapy has considered to be the main treatment. However, the emergence of drug resistance and the high rate of relapse of the neoplastic disease often lead to poor prognosis for SCLC patients, in spite of its initial high response to the chemotherapy [3]–[7], [9], [10]. A growing number of evidence suggests that biological markers may contribute greatly to the efficacy of chemotherapy; ERCC1 is one of the most notable biomarkers among them.

ERCC1 enzyme belongs to nucleotide excision repair (NER) system which has the ability of repairing DNA adducts and other DNA helix-distorting lesions [11], including platinum intra-strand DNA adducts, and it is considered to be related to resistance to platinum-based chemotherapy via reducing platinum-induced DNA damage [12]–[21]. Platinum mainly acts by binding to DNA and forming bulky DNA adducts, which caused inter-strand and intra-strand cross-link generation, as well as DNA-protein cross-links, resulting in inhibition of cell growth and apoptosis of targeted cells unless repaired. ERCC1 enzyme involved in recognizing and removing platinum-induced intra-strand adducts in DNA, and was reported to be a biomarker of resistance to platinum-based chemotherapy in patients with bladder, ovarian, colorectal, gastric, esophageal, and lung cancer [12]–[21].

The correlation between the efficacy of platinum-based chemotherapy in SCLC and ERCC1 expression has attracted wide-spread attention, and a lot of investigations have been conducted, whereas conflicting results were presented. Kim et al. [22] and Smit et al. [23] reported that there was no significant association between ERCC1 expression and overall response rate (ORR), progression-free survival (PFS) or overall survival (OS). On the other hand, Karachaliou et al [24] and Ceppi et al [21] demonstrated that low/negative expression of ERCC1 was related to longer OS. The relationship between ERCC1 expression and the clinical outcomes in SCLC patients receiving platinum-based chemotherapy thus remains unknown. Hence, we performed the present meta-analysis of all eligible investigations to derive a more precise evaluation of the association between ERCC1 level and the clinical outcomes (ORR, OS and PFS) in SCLC patients receiving platinum-based chemotherapy.

## Materials and Methods

### Search strategy

With the following terms combined: (“Excision Repair Cross-Complementation Group 1’’ or “ERCC1”) and (“small cell lung cancer” or SCLC or “neoplasm, lung”) and (“platinum” or “cisplatin” or “carboplatin” or “oxaliplatin”), we searched potentially relevant publications in online databases of PubMed, EMBASE and Web of Science for all years up until May 31, 2014. Only the papers written in English language were included. In addition, we also conducted an additional manual search on the basis of references.

### Inclusion and Exclusion Criteria

Eligibility criteria were as follows: (1) human-based studies; (2) pathologically confirmed SCLC receiving platinum-based regimens; (3) full-text written in English; (4) to evaluate the association between ERCC1expression and clinical outcomes, i.e. overall response rate (ORR), overall survival (OS) or progression-free survival (PFS). Exclusion criteria were as follows: (1) patients younger than 18 years old; (2) studies without necessary data to extract ORR, OS or PFS; (3) For overlapping data, the studies with small sample sizes or insufficient information were excluded.

### Data extraction and synthesis

Two independent researchers (X.P. Luo and Y.L. Yang) reviewed the included studies and extracted the following data: the first author, publication year, original country, ethnicity, sample size, age of patients, disease stage, chemotherapy regimens, detection method of ERCC1expression, distribution of high/positive ERCC1 expression in patients, and clinical outcomes. Any disagreement between the investigators was resolved through discussions until a consensus was reached.

### Quality assessment

The quality of the methodology of the included studies was assessed by the Newcastle-Ottawa scale (NOS) recommended by the Cochrane Non-Randomized Studies Methods Working Group [25]. Studies with five or more stars were defined as high quality studies. Quality assessment was performed by two investigators (Y.L. Yang and L. Xian) independently. Disagreements were resolved by discussion.

### Statistical analysis

The odds ratios (ORs) with corresponding 95% confidence intervals (CIs) were employed to estimate the association between ERCC1 status and the ORR to platinum-based chemotherapy in SCLC patients. The hazard ratios (HRs) and their corresponding 95% CIs were used to evaluate the relationship between ERCC1 expression and OS and PFS in SCLC patients treated by platinum-based chemotherapy. When the direct HRs and their 95% CIs were not provided in studies, the indirect estimates by Tierney's method were applied [26]. Heterogeneity among studies was evaluated by Q test and I^2^ metric [27]. In the presence of heterogeneity between studies (p<0.1 for Q test, or I^2^>50%), the pooled OR/HR was calculated using a random-effect model [28]; otherwise, the fixed-effect model was applied [29]. Subgroup analyses by ethnicity (Asian/Caucasian), stage of SCLC (LS/ES), and detection method (IHC/RT-PCR) were also conducted. Sensitivity analysis was performed to assess the stability of results. Each study was excluded one at a time to determine the magnitude of influence on the overall summary estimate. Publication bias was tested by funnel plots qualitatively and estimated by Begg's test and Egger's test quantitatively [30], [31]. All statistical analyses were performed by software of STATA version 11.2 (StataCorp, College Station, TX, USA).

## Results

### Eligible studies

**Figure 1:**
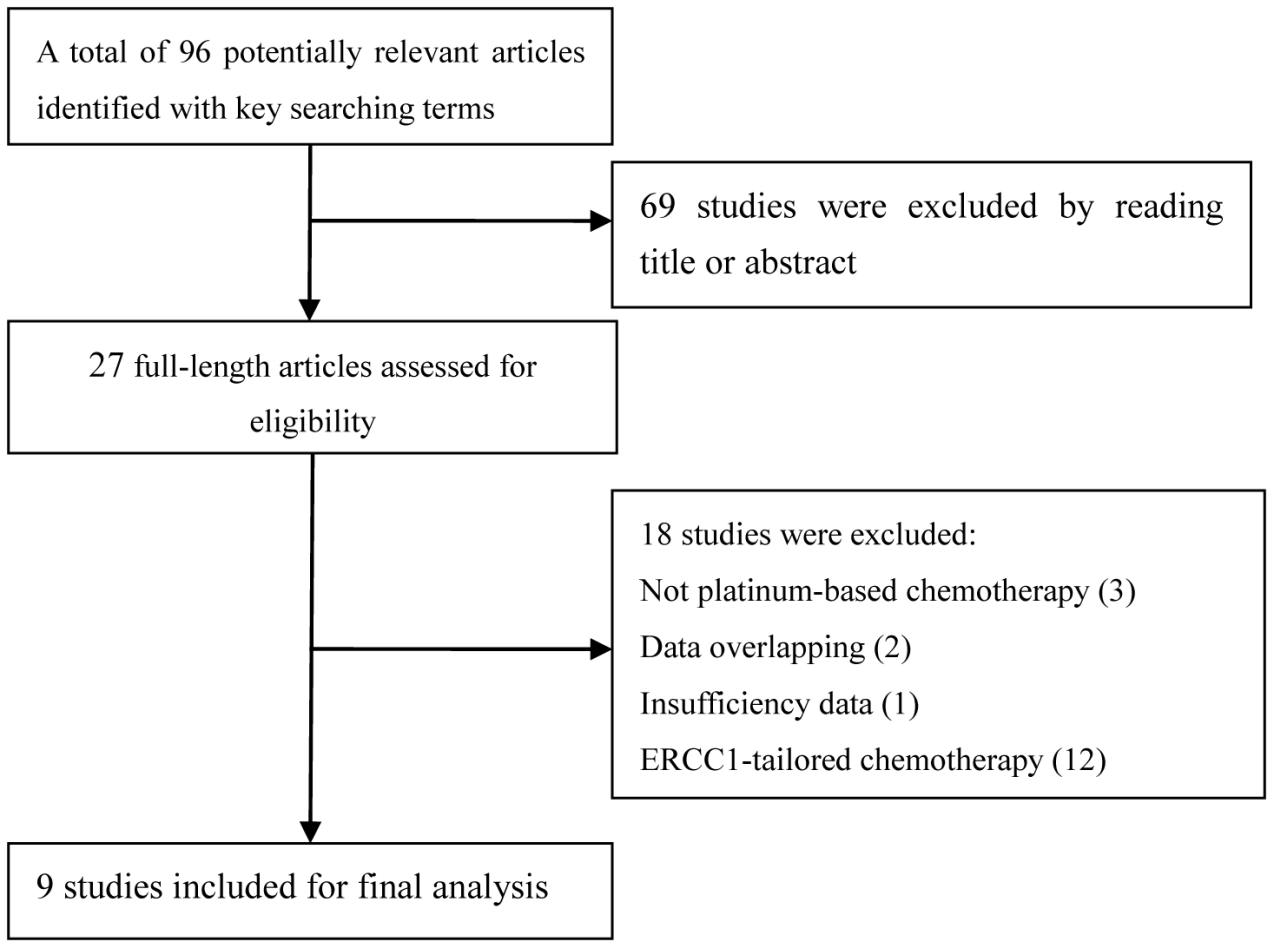
Flow diagram of the literature search in this meta-analysis.

The present work followed the guidelines for systematic reviews and meta-analyses (PRISMA) (Checklist S1). Details for the literature search were presented in Figure 1 and Figure S1. A number of 96 potentially relevant articles were yielded in the initial search with the searching terms. After reading the titles and abstracts, 27 potential articles were assessed for eligibility. However, 18 studies were further excluded because of the following reasons: not platinum-based chemotherapy (n = 3); data overlapping (n = 2); insufficiency data (n = 1); ERCC1-tailored chemotherapy (n = 12). Thus, nine studies including 1129 patients were included in meta-analysis [10], [21]–[24], [32]–[35]. All nine studies were assessed by the NOS quality scale and were scored highly (with five stars or more). The quality score of 9 eligible studies can be found in Table S1.

The characteristics of included publications were detailed in Table 1. The nine studies were published between 2008 and 2013. There were three studies conducted in Asian patients [10], [22], [32], five studies conducted in Caucasian patients [21], [24], [33]–[35], and one study conducted in mixed race [23]. The sample sizes of all the included studies ranged from 64 to 323. As shown in Table 1, in nine independent studies, one study focused on LS-SCLC [24], one study focused on ES-SCLC [23], and seven studies focused on both stage [10], [21], [32]–[35]. Of all eligible studies, three studies employed reverse transcription-polymerase chain reaction (RT-PCR) as detecting method [21], [24], [34] and the other six studies employed immunohistochemistry (IHC) [10], [22], [23], [32], [33], [35].

**Table 1:** Baseline characteristics of studies included in the meta-analysis.

Study	Year	Country	Ethnicity	No. of cases	Mean/median age	Stage	Therapy	Methods	Threshold	High expression (%)	Outcome
Ceppi[21]	2008	Italy	Caucasian	85	63(43–81)	LS, ES	Cisplatin/carboplatin and etoposide	RT-PCR	0.5	50.6	ORR,OS
Lee[10]	2008	Korea	Asian	77	61(41–76)	LS, ES	Platinum-based doublets	IHC	H-score = 2	22.1	ORR,OS
Kim[22]	2009	Japan	Asian	130	67(28–83)	ES, LS	Platinum-based combination	IHC	10%	27.7	ORR,OS
Skov[33]	2010	Denmark	Caucasian	186	68 (47–93)	LS, ES	Platinum-based combination	IHC	H-score >0	9.1	OS
Smit[23]	2011	NR	Mixed	323	NR	ES	Platinum-based combination	IHC	H-score = 25	27.7	OS
Lee[32]	2012	Korea	Asian	111	65 (44–80)	LS, ES	Platinum-based doublets	IHC	H-score = 2	44.1	ORR,OS
Sereno[34]	2012	Spain	Caucasian	76	62(43–81)	LS, ES	Etoposide-cisplatin, carboplatin-etoposide	RT-PCR	0.1	54.7	ORR,PFS
Sodja[35]	2012	Slovenia	Caucasian	77	59 (42–77)	LS, ES	Platinum-based doublets	IHC	50%	51.9	ORR,OS,PFS
Karachaliou[24]	2013	Greece	Caucasian	64	63 (33–78)	LS	Cisplatin-etoposide.	RT-PCR	Median(11.49)	50.0	OS,PFS

**Abbreviations:** **NR**, not reported; **LS**, limited stage; **ES**, extensive stage; **IHC**, immunohistochemistry; RT-**PCR**, reverse transcription-polymerase chain reaction; **PFS**, progression-free survival;

**ORR**, objective response rate; **OS**, overall survival.

### Objective response rate

**Figure 2:**
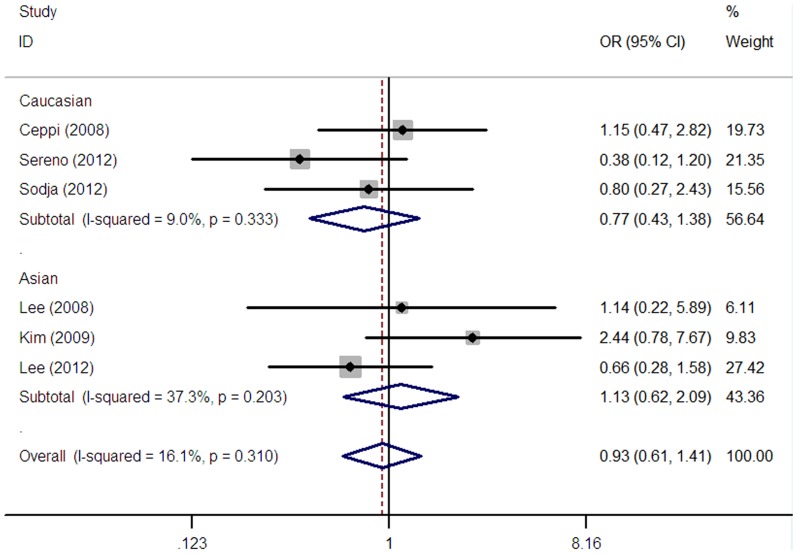
Forest plot for the association between ERCC1 level and objective response rate (ORR) in SCLC patients receiving platinum-based chemotherapy.

The association between ERCC1 expression and the response to platinum-based chemotherapy was explored in six studies containing 533 SCLC patients [10], [21], [22], [32], [34], [35]. And the result indicated that ERCC1 expression was not significantly associated with the ORR in SCLC patients receiving platinum-based chemotherapy (OR = 0.93, 95% CI = 0.61–1.41, I^2^ = 16.1%, P = 0.31 for heterogeneity; Table 2, Figure. 2).

**Table 2:** Meta-analysis results about ERCC1 expression and ORR, OS and PFS in SCLC patients who received platinum-based chemotherapy.

	ORR	OS	PFS
	Study (No. of patients)	Model	OR(95%CI)	I^2^,P	Study (No. of patients)	Model	HR(95%CI)	I^2^,P	Study (No. of patients)	Model	HR(95%CI)	I^2^,P
Overall	6(533)	F	0.93(0.61–1.41)	16.1%,0.31	**8(1053)**	**F**	1.18(1.02–1.37)	**19.8%,0.27**	**3(217)**	**F**	**1.46(1.14–1.88)**	**15.1%,0.31**
Ethnicity												
Asian	3(318)	F	1.13(0.62–2.09)	37.3%,0.20	3(318)	F	1.25(0.96–1.63)	47.4%,0.13	/	/	/	/
Caucasian	3(215)	F	0.77(0.43–1.38)	9.0%,0.33	**4(412)**	**F**	1.25(1.01–1.53)	**0%,0.41**	/	/	/	/
Stage												
LS	3(106)	F	1.66(0.61–4.48)	21.0%,0.28	**5(210)**	**F**	1.54(1.21–1.96)	**38.2%,0.17**	**2(96)**	**R**	**1.39(1.01–1.91)**	**0.0%,0.55**
ES	3(167)	F	0.67(0.35–1.29)	39.9%,0.19	4(487)	F	0.96(0.74–1.24)	0.0%,0.74	1(45)	/	1.02(0.55–1.89)	**/**
Detection method												
IHC	4(395)	F	1.05(0.671,1.78)	11.7%,0.33	6(886)	F	1.10(0.91,1.31)	24.4%,0.24	1(77)	/	1.32(0.82.2.15)	/
RT-PCR	2(138)	R	0.71(0.24,2.05)	54.3%,0.139	**2(167)**	**F**	1.37(1.06,1.78)	**0.0%,0.74**	**2(140)**	**R**	**1.63(1.00,2.63)**	**53.1%,0.14**

**Abbreviations: ERCC1**, excision repair cross complementation group 1; **SCLC**, small cell lung cancer; **LS**, limited stage; **ES**, extensive stage; **R**, random model; **F**, fixed model; **ORR**, objective response rate; **OS**, overall survival; **PFS**, progression-free survival.

Subgroup analyses according to ethnicity and disease stage were also performed in this meta-analysis. Of the six studies reporting the data on ORR, three studies [10], [22], [32] consisted of 318 patients were conducted among Asians and the other three studies [21], [34], [35] containing 215 patients were performed among Caucasians. Subgroup analysis based on ethnicity indicated that ERCC1 expression was not related to the ORR to platinum-based chemotherapy for SCLC patients both among Asian (OR = 1.13, 95%CI = 0.62–2.09, I^2^ = 37.3%, P = 0.20 for heterogeneity) (Table 2) and Caucasian population (OR = 0.77, 95%CI = 0.43–1.38, I^2^ = 9.0%, P = 0.33 for heterogeneity) (Table 2). Three studies focused on limited stage [21], [32], [35], Three studies focused on extensive stage [21], [32], [35]. Subgroup analysis on the basis of disease stage suggested that ERCC1 expression was not associated with the ORR to platinum-based chemotherapy in limited stage (OR = 1.66, 95%CI = 0.61–4.48, I^2^ = 21.0%, P = 0.28 for heterogeneity) or extensive stage (OR = 0.67, 95%CI = 0.35–1.29, I^2^ = 39.9%, P = 0.19 for heterogeneity). According to ERCC1 detection method, both IHC and RT-PCR groups did not show significant association between the expression of ERCC1 and the ORR in patients with SCLC (Table 2).

### Overall survival

**Figure 3:**
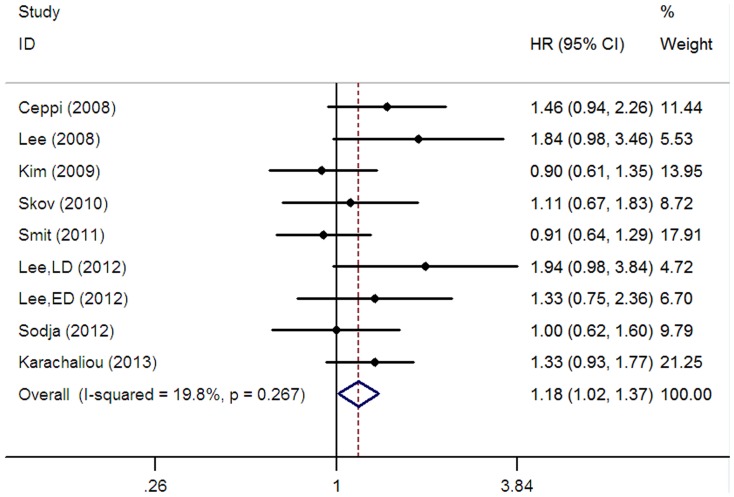
Forest plot for the association between ERCC1 level and overall survival (OS) in SCLC patients receiving platinum-based chemotherapy.

**Figure 4:**
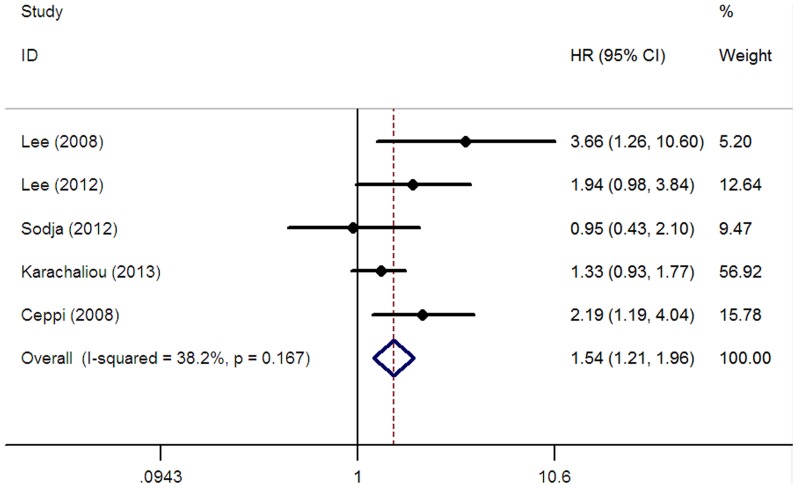
Forest plot for the association between ERCC1 level and overall survival (OS) in LS-SCLC patients receiving platinum-based chemotherapy.

A total of eight studies [10], [21]–[24], [32], [33], [35] composed of 1053 patients reported the data on overall survival (OS). Overall, the pooled analysis showed that positive/high ERCC1 expression was significantly associated with shorter OS for SCLC patients receiving platinum-based chemotherapy (HR = 1.18, 95% CI = 1.02–1.37, I^2^ = 19.8%, P = 0.27 for heterogeneity; Table 2, Figure 3). Of the eight studies reporting the data on OS, three studies [10], [22], [32] were conducted in Asian patients, four studies [21], [24], [33], [35] were conducted in Caucasians and one study [23] was conducted in mixed ethnicity. In the stratified analysis according to ethnicity, our analysis indicated that ERCC1 expression was related to OS in Caucasian population (HR = 1.25, 95% CI = 1.01–1.53, I^2^ = 0.0%, P = 0.41 for heterogeneity; Table 2), but not in Asian population (HR = 1.25, 95% CI = 0.96–1.63, I^2^ = 47.4%, P = 0.13 for heterogeneity; Table 2). Five studies [10], [21], [24], [32], [35] focused on limited stage, Four studies [10], [23], [32], [35] focused on extensive stage. Subgroup analysis based on disease stage suggested that the patients with positive/high ERCC1 expression would have shorter OS for limited stage (positive/high vs. negative/low: HR = 1.54, 95% CI = 1.21–1.96, I^2^ = 38.2%, P = 0.17 for heterogeneity; Table 2, Figure 4), but not for extensive stage (positive/high vs. negative/low: HR = 0.96, 95% CI = 0.74–1.24, I^2^ = 0.0%, P = 0.74 for heterogeneity; Table 2). We then focused on ERCC1 detection method, only two studies [21], [24] using RT-PCR showed a significant association between the expression of ERCC1 and the OS in patients with SCLC (HR = 1.37, 95% CI = 1.06–1.78, I^2^ = 0.0%, P = 0.74 for heterogeneity; Table 2).

### Progression-free survival

**Figure 5:**
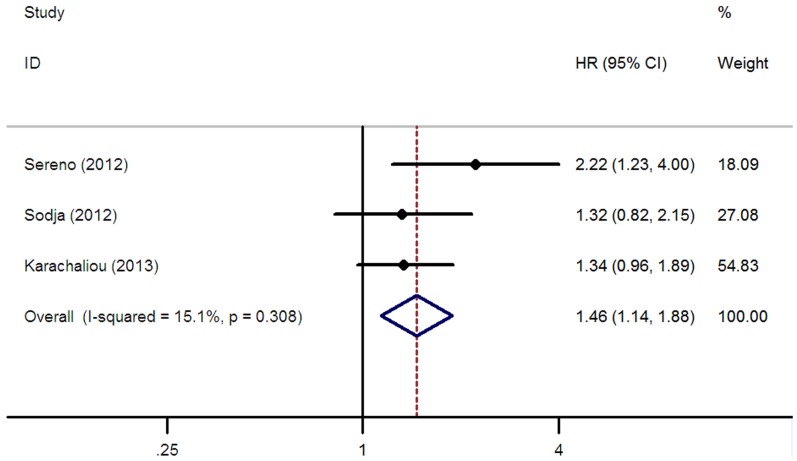
Forest plot for the association between ERCC1 level and progression free survival (PFS) in SCLC patients receiving platinum-based chemotherapy.

The association of ERCC1 expression and PFS was explored in three studies [24], [34], [35] composed of 217 SCLC patients. The summarized analysis showed that positive/high ERCC1 expression was associated with unfavorable PFS for SCLC patients receiving platinum-based chemotherapy (HR = 1.46, 95% CI = 1.14–1.88, I^2^ = 15.1%, P = 0.31 for heterogeneity; Table 2, Figure 5). In the stratified analysis according to disease stage, significant association between ERCC1 expression and PFS was found in limited stage (HR = 1.39, 95% CI = 1.01–1.91, I^2^ = 0.0%, P = 0.55 for heterogeneity; Table 2), but not in extensive stage (HR = 1.02, 95% CI = 0.55–1.89; Table 2). The subgroup analysis of the ERCC1 detection methods indicated that the expression of ERCC1 was significantly associated with the PFS in the two studies [24], [34] using RT-PCR as the detection method (HR = 1.63, 95% CI = 1.00–2.63, I^2^ = 53.1%, P = 0.14 for heterogeneity; Table 2).

### Sensitivity analysis

In the sensitivity analysis, the influence of each study on the pooled OR/HR was examined by repeating the meta-analysis while omitting each study one at a time. When the study by Karachaliou et al. [24] was excluded, the relationship between ERCC1 expression and OS in overall SCLC patients showed no significant association (HR = 1.14, 95% CI = 0.97–1.35, I^2^ = 24.7%, P = 0.23 for heterogeneity), indicating the study by Karachaliou et al.may affect the OS in overall patients. Except for OS, the analysis results suggested that no individual study significantly affected the pooled OR/HR in ORR and PFS.

### Publication bias

Visual inspection of the funnel plot did not show any evidence of obvious asymmetry in three comparisons (figure not shown). Also, no significant bias was indicated by the Begg's and Egger's test (ORR: P_Begg_ = 0.71, P_Egger_ = 0.78; OS: P_Begg_ = 0.05, P_Egger_ = 0.14; PFS: P_Begg_ = 0.30, P_Egger_ = 0.44).

## Discussion

In the present meta-analysis, our analysis indicated that SCLC patients with positive/high ERCC1 expression were significantly associated with shorter OS and PFS, especially in LS-SCLC. Hoverer, no significant association between ERCC1 expression and ORR was observed.

Previous meta-analysis by Hubner et al. [36] has reported that ERCC1 expression was not associated with OS for SCLC patients receiving platinum-based chemotherapy. In the stratified analysis according to stage, they found ERCC1 expression was associated with OS in LS-SCLC, but not in ES-SCLC. In the present study, we observed that positive/high ERCC1 expression was related to shorter OS and PFS. In subgroup analysis by stage, we found high/positive ERCC1 was associated with worse OS and PFS in LS-SCLC, but not in ES-SCLC. We think it is necessary to update the meta-analysis and make a more comprehensive estimation of the association between ERCC1 expression and the efficacy of platinum-based chemotherapy for the following reasons. Firstly, compared with the study by Hubner et al., which containing 292 patients from five studies, our analysis included larger sample size of 1129 patients from 9 studies, with which the reliability was largely enhanced. Besides, Hubner et al only evaluated the relationship between ERCC1 expression and OS; in the present analysis, we employed ORR, OS and PFS as primary parameters to comprehensively examine the relationship between ERCC1 expression and clinical outcomes of platinum-based chemotherapy. As we all know, a low ORR indicates tumor resistance to the chemotherapeutic regimen and a short OS and PFS suggest a poor prognosis. Thus, including all three parameters could make a comprehensive assessment. However, our analysis did not find the significant association between ERCC1 expression and ORR.

The weight of the study by Karachaliou et al. [24] is considerable in figures 3(21.25%), 4 (56.92%) and 5(54.83%). We think three possible reasons should be acknowledged. First of all, the sample size is relatively larger. A total of 64 LS-SCLC patients were included. In the comparison of OS in LS-SCLC (figure 4), and PFS in overall patients (figure 5), the patients' number in this study account for 30.5% and 29.5% in overall patients, respectively. Secondly, the study by Karachaliou et al is highly homogeneous. All included samples were LS-SCLC, they all received first line cisplatin-etoposide doublet. In other studies, most of studies received all kinds of platinum-based doublets and may be biased by imbalances between studies regarding fist-, second- and later-lines of treatment. At last, the HRs we obtained from this study were multivariate-adjusted results. In the sensitivity analysis, when the study by Karachaliou et al was removed in each comparison, except for the overall OS, the HR for OS in LS-SCLC and the overall PFS did not changed (for overall OS, HR = 1.14, 95% CI = 0.97–1.35; for OS in LS-SCLC, HR = 1.87, 95% CI = 1.29–2.71; for overall PFS, HR = 1.63, 95% CI = 1.12–2.36, respectively). As many study found the ERCC1 expression was more likely to be a prognostic factor in limited stage-SCLC, it was not surprise that the HR in overall OS was not significant when the study by Karachaliou et al (all included samples were LS-SCLC) was removed. Generally, the result of our study was stable and reliable.

Why the significant association between positive/high ERCC1 expression and shorter OS and PFS was mainly found in patients with LS-SCLC? Firstly, the possibility is that disease progression from the limited to the extensive stage leads to a potential transcriptional deregulation of ERCC1 for SCLC patients [21]. In the study by Kang et al., who found when compared with primary tumors in NSCLC, ERCC1 expression was upregulated in metastatic lymph nodes [37]. Besides, the expression of ERCC1 would be induced by platinum. In human lung adenocarcinoma cell line A549, up-regulation of ERCC1 expression could be induced by low-dose cisplatin [38]; in metastatic colorectal cancer, after oxaliplatin-based first-line chemotherapy, ERCC1 expression was upregulatedin [39]. Also, the standard treatment for patients with LS-SCLC is a combination of concurrent thoracic irradiation and chemotherapy with etoposide and cisplatin whereas for patients with ES-SCLC, chemotherapy is the main method. ERCC1 is related to radiation-induced DNA damage in a few in-vitro studies, although the mechanism is not well known [40], [41]. Thus, this could probably be the consequence of expression levels up-regulation resulting in radiotherapy. At last, patients with high expression of ERCC1 might benefit from alternative strategies such as incorporation of different sequencing of chemoradiotherapy and new chemotherapeutic agents [3]–[5], which may lead to false positive. However, the specific regulatory mechanism need to be further investigated in the future.

It was meaningful to identify more efficacy biomarkers for SCLC, especially for ES-SCLC. Many biomarkers have been evaluated, for example, multidrug resistance-associated proteins such as multidrug resistance protein 1 (P-glycoprotein; MDR1), multidrug resistance-associated protein1 (MRP1) and 2 (MRP2), major vault protein (MVP), topoisomerases II (topo II) [42], and serum markers such as lactate dehydrogenase (LDH), neuron-specific enolase (NSE) [43]and so on. Nowadays, circulating tumour cells (CTCs) has also emerged as a potential predictive and prognostic factor for SCLC [44]. As the predictive and prognostic role of a single biomarker is limited, to detect more relevant biomarkers as a combination would be more efficient.

However, a clinical value of the findings obtained by our meta-analysis remains questionable. Friboulet L. et al. was unable to validate the predictive effect of immunostaining for ERCC1 protein by three independent trails [IALT, Cancer and Leukemia Group B (CALGB) 9633, and National Cancer Institute of Canada Clinical Trials Group JBR.10 trials] [45]. This may have arisen from assessment of ERCC1 expression in clinical samples is complicated by the existence of 4 functionally distinct protein isoforms (201, 202, 203, and 204); on the other hand, current available antibodies used to evaluate ERCC1 expression do not have adequate discrimination. In our study, the included studies detected ERCC1 by different method: three studies detected ERCC1 expression in transcriptional level by RT-PCR, and six studies determined at protein level by IHC. In addition, the detecting tools and the cutoff values for ERCC1 expression were different from each other. Friboulet's study also supported the evidence that the ERCC1-202 isoform is the sole one endowed with ERCC1 activity in DNA repair pathways and might support as an accurate predictor of cisplatin benefit in patients with NSCLC [45]. Further studies were wanted to elucidate the predict role of ERCC1-202 isoform in platinum-based chemotherapy.

Several limitations should be addressed when interpreting the result of our meta-analysis. First, the individual studies included in our study differed obviously in the study design, such as ERCC1 detection method, patient selection and chemotherapeutic protocols. Our meta-analysis may be biased by sex, age, smoking status, different detection method for ERCC1, specific platinum-based drug employed (e.g., cisplatin, carboplatin, or oxaliplatin), and the imbalances between studies regarding fist-, second- and later-lines of treatment, which could influence the homogeneity among the individual studies. Secondly, apart from the chemotherapy, some studies also had surgery or radiotherapy, while the others did not have the therapy. Thirdly, due to lack of sufficient data regarding confounding factors, such as sex, age and smoking status from the original studies, we did not perform stratified analysis to evaluate the effects of these confounding factors on the predictive effect of ERCC1 for SCLC patients. Besides, the sample size in our analysis was not large enough. Only six studies including 533 patients investigated the relationship between ERCC1 expression and ORR, and three studies including 217 patients evaluated the PFS, such limited sample size still could not draw a robust conclusion and need to be interpreted with caution, Finally, potential publication bias may remain because the databases we searched and resources we processed were limited and the publications written not in English or with negative results which failed to be published were failed to be included in the present analysis.

In conclusion, this meta-analysis provided the evidence that SCLC patients with positive/high ERCC1 would have worse OS and PFS, especially in patients with LS-SCLC. Therefore, ERCC1 might act as a valuable marker of prognosis in patients with SCLC treated by platinum-based chemotherapy. However, considering the limitations and confounds of the present study, our conclusions need to be interpreted with caution. Large prospective studies with strict designed methodology are warranted to further investigated the prognostic role of ERCC1 in SCLC patients receiving platinum-based treatment.

## Supporting Information

Figure S1 PRISMA flow diagram.
(DOC)

Table S1 Quality assessment of eligible studies with Newcastle-Ottawa Scale.
(DOCX)

Checklist S1 PRISMA checklist.
(DOC)
