# Supplementary material for: The Prognostic Value of Excision Repair Cross-Complementation Group 1 (ERCC1) in Patients with Small Cell Lung Cancer (SCLC) Receiving Platinum-Based Chemotherapy: Evidence from Meta-Analysis
Source: PLoS One. 2014 Nov 6;9(11):e111651. doi: 10.1371/journal.pone.0111651 (PMC4222940; doi:10.1371/journal.pone.0111651)
Supplement: Figure S1 — PRISMA flow diagram. (DOC) [file pone.0111651.s001.doc]

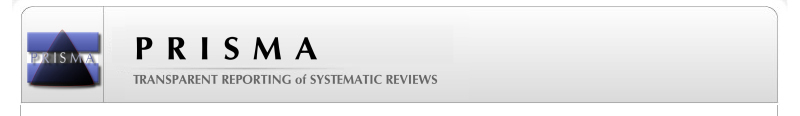
**PRISMA 2009 Flow Diagram**

**Screening**

**Included**

**Eligibility**

**Identification**

Records identified through database searching
(n =96 )

Additional records identified through other sources
(n =0 )

Records after duplicates removed
(n = 96 )

Records screened
(n = 96 )

Records excluded
(n = 69 )

Full-text articles assessed for eligibility
(n = 27 )

Full-text articles excluded, with reasons
(n = 18 )

Studies included in qualitative synthesis
(n =9 )

Studies included in quantitative synthesis (meta-analysis)
(n = 9 )
