# Supplementary material for: The Prognostic Value of Excision Repair Cross-Complementation Group 1 (ERCC1) in Patients with Small Cell Lung Cancer (SCLC) Receiving Platinum-Based Chemotherapy: Evidence from Meta-Analysis
Source: PLoS One. 2014 Nov 6;9(11):e111651. doi: 10.1371/journal.pone.0111651 (PMC4222940; doi:10.1371/journal.pone.0111651)
Supplement: Table S1 — Quality assessment of eligible studies with Newcastle-Ottawa Scale. (DOCX) [file pone.0111651.s002.docx]

Table S1.Quality assessment of eligible studies with Newcastle-Ottawa Scale

| Author | Year | Selection | Comparability | Outcome |
| --- | --- | --- | --- | --- |
| Ceppi | 2008 | ★★★ | ★★ | ★★★ |
| Lee | 2008 | ★★ | ★★ | ★★ |
| Kim | 2009 | ★★★ | ★★ | ★★ |
| Skov | 2011 | ★★ | ★★ | ★★ |
| Smit | 2011 | ★★★ | ★ | ★★ |
| Lee | 2012 | ★★★ | ★★ | ★★ |
| Sereno | 2012 | ★★ | ★ | ★★ |
| Sodja | 2012 | ★★★ | ★★ | ★★ |
| Karachaliou | 2013 | ★★★ | ★★ | ★★★ |
